# An update and ecological perspective on certain sentinel helminth endoparasites within the Mediterranean Sea

**DOI:** 10.1017/S0031182023000951

**Published:** 2023-10

**Authors:** Marialetizia Palomba, Erica Marchiori, Perla Tedesco, Marialetizia Fioravanti, Federica Marcer, Andrea Gustinelli, Renato Aco-Alburqueque, Beatrice Belli, Daniele Canestrelli, Mario Santoro, Paolo Cipriani, Simonetta Mattiucci

**Affiliations:** 1 Department of Ecological and Biological Sciences (DEB), Tuscia University, Viterbo, Italy; 2 Department of Animal Medicine, Production and Health, University of Padova, Legnaro, Padua, Italy; 3 Department of Veterinary Medical Sciences, University of Bologna, Ozzano dell'Emilia, Bologna, Italy; 4 Department of Public Health and Infectious Diseases, Section of Parasitology, Sapienza University of Rome, Rome, Italy; 5 Department of Integrative Marine Ecology, Stazione Zoologica Anton Dohrn, Naples, Italy; 6 Section of Contaminants and Biohazards, Institute of Marine Research (IMR), Nordnes, Bergen, Norway

**Keywords:** anthropogenic changes, Ascaridoidea, ecological indicators, Mediterranean Sea, Oncoproteocephalidea, Tetraphyllidea, trophically transmitted helminths, Trypanorhyncha

## Abstract

The Mediterranean Sea is recognized as a marine biodiversity hotspot. This enclosed basin is facing several anthropogenic-driven threats, such as seawater warming, pollution, overfishing, bycatch, intense maritime transport and invasion by alien species. The present review focuses on the diversity and ecology of specific marine trophically transmitted helminth endoparasites (TTHs) of the Mediterranean ecosystems, aiming to elucidate their potential effectiveness as ‘sentinels’ of anthropogenic disturbances in the marine environment. The chosen TTHs comprise cestodes and nematodes sharing complex life cycles, involving organisms from coastal and marine mid/upper-trophic levels as definitive hosts. Anthropogenic disturbances directly impacting the free-living stages of the parasites and their host population demographies can significantly alter the distribution, infection levels and intraspecific genetic variability of these TTHs. Estimating these parameters in TTHs can provide valuable information to assess the stability of marine trophic food webs. Changes in the distribution of particular TTHs species can also serve as indicators of sea temperature variations in the Mediterranean Sea, as well as the bioaccumulation of pollutants. The contribution of the chosen TTHs to monitor anthropogenic-driven changes in the Mediterranean Sea, using their measurable attributes at both spatial and temporal scales, is proposed.

## Graphical abstract

**Figure:**
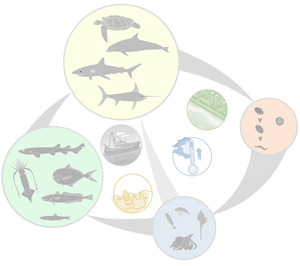

## Introduction

The Mediterranean Sea, despite occupying only 0.8% of the global ocean's surface, is recognized as a hotspot of biodiversity, hosting around 7.5% of the world marine species (Coll *et al.*, 2010). Within this species richness, the Mediterranean ecosystems also hide an important component of the marine environment, represented by trophically transmitted helminth endoparasites (TTHs). These organisms constitute a dominant community of both the pelagic and benthic food webs. Several digenean trematodes, cestodes, nematodes and acanthocephalans, previously listed (Euzet, 1959; Petter and Radujkovic, 1989; Bona *et al.*, 1995; Paggi, 2008; Radujkovic and Sundic, 2014), constitute the TTHs biodiversity of Mediterranean Sea. However, although recent decades have seen a considerable progress in the taxonomic classification of marine parasites through an integrative approach, involving both morphological and molecular methods, a significant number of TTHs species still remains unidentified, and our knowledge of their ecology is incomplete.

This review focuses on the current state of knowledge on the biodiversity and ecology of certain TTHs among nematodes and cestodes from the Mediterranean Sea, whose taxonomic status has been assessed by both morphological and genetic/molecular methodologies. The chosen TTHs exhibit complex life cycles, with free-living phases and use of several host organisms at different trophic levels. Crustaceans, molluscs and fish can act as intermediate and paratenic hosts, while large organisms of mid to high trophic levels, such as cetaceans, sea turtles, large teleosts and elasmobranchs, are definitive hosts.

The Mediterranean ecosystem faces a wide range of anthropogenic stressors, such as global warming, overexploitation of fish resource, bycatch, intense maritime transport, eutrophication and land-based pollutants/contaminants (Mandić and Piraino, 2023). There is increasing evidence that these stressors dramatically affect the demography, migration patterns and spawning sites of marine organisms, including the hosts involved in the life cycle of the TTHs here considered. Consequently, these changes could also impact the dynamics of parasite populations (Marcogliese, 2005; Lafferty *et al.*, 2008; Timi and Poulin, 2020). Understanding the situation and dynamics of TTHs–host associations in the Mediterranean basin can offer a further perspective in the monitoring of the overall ‘health status’ of this marine environment, and estimate its vulnerability to various human-induced pressures.

We first present the rationale behind choosing certain groups of TTHs as ‘sentinel’ species. Then, by examining the ecological aspects of the chosen TTHs, the review critically evaluates their significance as indicators of trophic web functioning and stability in the Mediterranean Sea. Indeed, being intimately related to trophic transmission and specific to their hosts, these helminth endoparasites require stable trophic webs, as well as a wide range and population size of suitable hosts to complete their life cycles (Marcogliese, 2005; Mattiucci and Nascetti, 2008). Any anthropogenic stressor that could lead to decline in the host populations of the chosen TTHs could also impact the population size of their endoparasites (Marcogliese, 2005). As a consequence, a loss of genetic variability can occur in the parasites' gene pool as the result of genetic drift phenomena (Bullini *et al.*, 1986; Mattiucci and Nascetti, 2008). In this regard, the review aims to provide examples of temporal and spatial variations in infection levels and genetic variability of some among the chosen TTHs. Estimates of parasitic load and genetic variability of some TTHs are here proposed as indirect indicators of trophic web stability of marine ecosystem.

Furthermore, the host–parasite associations of the chosen TTHs can be significantly influenced by abiotic environmental factors, which are impacted by ongoing climate change (Palm, 2011). Indeed, the rising sea levels and increasing temperatures in Mediterranean waters (Marriner *et al.*, 2022) can trigger shifts in water circulation and variation in salinity. These, among other abiotic perturbations, can directly affect the hatching of eggs and the first free-living larval stage of TTHs, as well as influence the presence and abundance of first intermediate hosts (i.e. invertebrates) of these parasites. These same environmental perturbations can also alter the migratory routes for feeding and/or reproduction of both intermediate/paratenic and definitive hosts of TTHs. In this context, this study aims to highlight the role of some TTHs as an ecological tool for monitoring the consequences of warming Mediterranean marine environment. Finally, the review aims to provide examples, so far existing, where TTHs would serve as environmental indicators of the impact of pollutants originating from offshore sea activities and coastal areas.

## What are criteria to choose certain TTHs as ‘sentinel’ parasites?

To date, the use of marine parasites as biological indicators in Mediterranean waters has primarily centred on their linkage to fisheries. In this context, distribution and relative proportions of parasite species with known geographical ranges have been employed as part of a holistic approach, as biological tags for identifying fish stocks (Palm, 2011; Timi and Mackenzie, 2015; Mattiucci *et al.*, 2015*a*). Indeed, some TTHs (i.e. *Anisakis* spp. and trypanorhynch larvae) proved to be valid indicators of distinct Mediterranean biological and genetic stocks as opposed to those from the NE Atlantic waters, of pelagic and demersal fish species, such as the European hake *Merluccius merluccius*, the Atlantic horse mackerel *Trachurus trachurus*, the swordfish *Xiphias gladius* (reviewed in Mattiucci et al., 2015*a*) and the European pilchard *Sardina pilchardus* (Caballero-Huertas *et al.*, 2022). In the use of parasites as tags, phylogeographic aspects of the parasite species have also been included to determine fish host populations (Mattiucci *et al.*, 2018*a*). However, comparatively, less emphasis has been spent on exploring the potential use of marine TTHs as indicators of trophic web stability and ecosystem functioning.

The TTHs here considered were chosen based on the following criteria: (1) being endemic or widely distributed in the Mediterranean Sea. They should be easily visible and collectable, with documented presence in Mediterranean regions and have been reported in scientific literature or studied within the past 2 decades, in order to follow, whenever possible, their population dynamics; (2) having a known life cycle in its general pathway. The chosen parasite species should have, at least, confirmed definitive and/or intermediate/paratenic hosts inhabiting the Mediterranean Sea. Further, it should be easily identifiable at the trophic levels (feeding relationships) of the host species involved in the parasite's life cycle; (3) being specialist rather than generalist species *vs* definitive hosts: i.e. the parasitic species should display a preference for definitive hosts, rather than infecting a wide range of host species not ecologically correlated; (4) not having a high pathogenic role to their hosts; (5) being common in their definitive host or/and intermediate/paratenic hosts. Their density should have been studied, possibly, even at spatial scale; (6) being well-characterized at both the morphological and molecular level. The chosen species should have been studied by an integrative approach, including both morphological and genetic methodologies, allowing their clear recognition; (7) whenever possible, having access to historical comparative data on their occurrence in Mediterranean waters.

## What are the most reported TTHs from organisms of mid-high trophic levels?

The existing literature consistently highlights that the predominant taxa of TTHs in Mediterranean waters, aligning with those specified indicator criteria, includes nematodes and cestodes. In the last decades, these helminths have attracted scientific attention due to their constant presence, mainly at their larval stage, in commercially important marine organisms, such as fish and molluscs, from the Mediterranean Sea (Cancellieri *et al.*, 2023). Extensive studies of certain TTHs have been motivated by the zoonotic role played by some nematode species (i.e. *Anisakis pegreffii*, Campana-Rouget and Biocca, 1955; see Nascetti *et al.*, 1986) (reviewed in Mattiucci *et al.*, 2018*b*), which are widespread in Mediterranean wild fisheries. Other TTHs such as larval *Hysterothylacium*, *Sulcascaris* and/or trypanorhynch cestodes, even though not considered zoonotic, could by their presence make seafood unappealing to the consumer, thereby compromising the quality and value of the products (Bao *et al.*, 2020; Rudders *et al.*, 2023). Sporadic reports of human infections by *Hysterothylacium aduncum* (Rudolphi, 1802) Deardorff and Overstreet, 1981 exist in literature (Yagi *et al.*, 1996). However, in these records, pathological findings and molecular identification of the recovered parasite were not described. Similarly, metacestodes of the chosen TTHs are not considered zoonotic parasites. However, there is speculation that Trypanorhyncha larvae, i.e. *Gymnorhynchus gigas* (Cuvier, 1817) Rudolphi, 1819 and *Molicola horridus* (Goodsir, 1841) Dollfus, 1935, invading fish host muscles, might release proteins triggering allergic reactions in humans (Gòmez-Morales *et al.*, 2008). Although these proteins have not yet been identified in marine metacestodes, it is generally known that proteins from zoonotic larval helminths involved in host tissue invasion, such as in the infection by *A. pegreffii*, could lead to IgE-hypersensitization in the immune response of accidental human hosts (Mattiucci *et al.*, 2017*a*; Palomba *et al.*, 2019, 2023*a*).

In addition, over the last 30 years, the implementation of molecular epidemiological surveys on these TTHs, carried out in wild fisheries, bycatches and stranded animals along the Mediterranean coasts, has substantially enhanced our knowledge regarding their ecological aspects. This includes life cycles, host range and geographical distribution within Mediterranean waters (Palm *et al.*, 2009; Mattiucci *et al.*, 2018*b*, 2019; Marcer *et al.*, 2020). Many TTHs species rely on cetaceans, sea turtles, elasmobranchs and large teleostean fish as definitive hosts. Studies on these parasites in such ‘charismatic’ hosts have drawn particular attention, since these hosts are recognized as ‘flagship species’, many of which are endangered, protected or subject to specific conservation measures.

**Figure 1.**
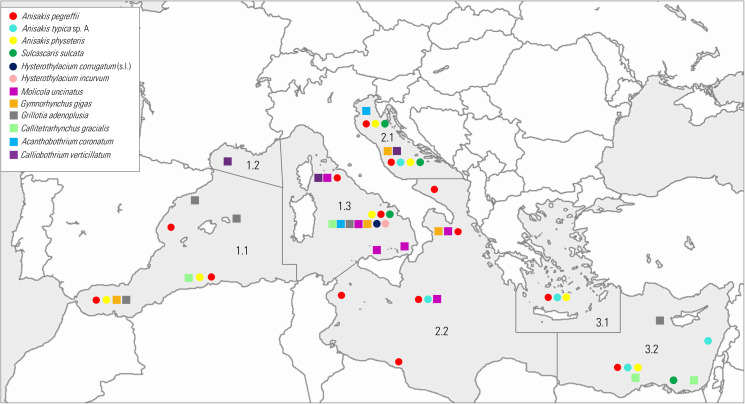
Trophically transmitted helminth endoparasite (TTHs) distributions mapped into the FAO (major fishing regions) (area 37) of the Mediterranean Sea.

**Figure 2.**
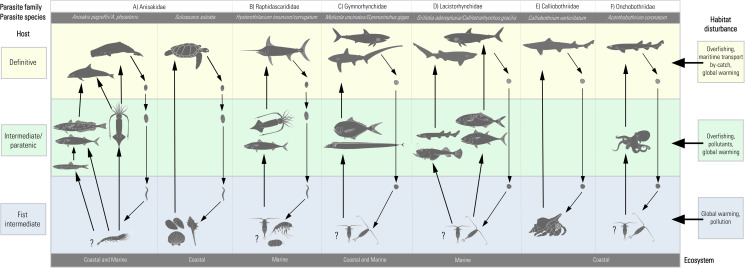
Schematic representation of the life cycle of the TTHs here considered. (A) Anisakidae; (B) Raphidascarididae; (C) Gymnorhynchidae; (D) Lacistorhynchidae; (E) Calliobothriidae; (F) Onchobothriidae. On the right, anthropogenic stressors, affecting the trophic web levels through which the TTHs move their life cycle, are reported.

**Figure 3.**
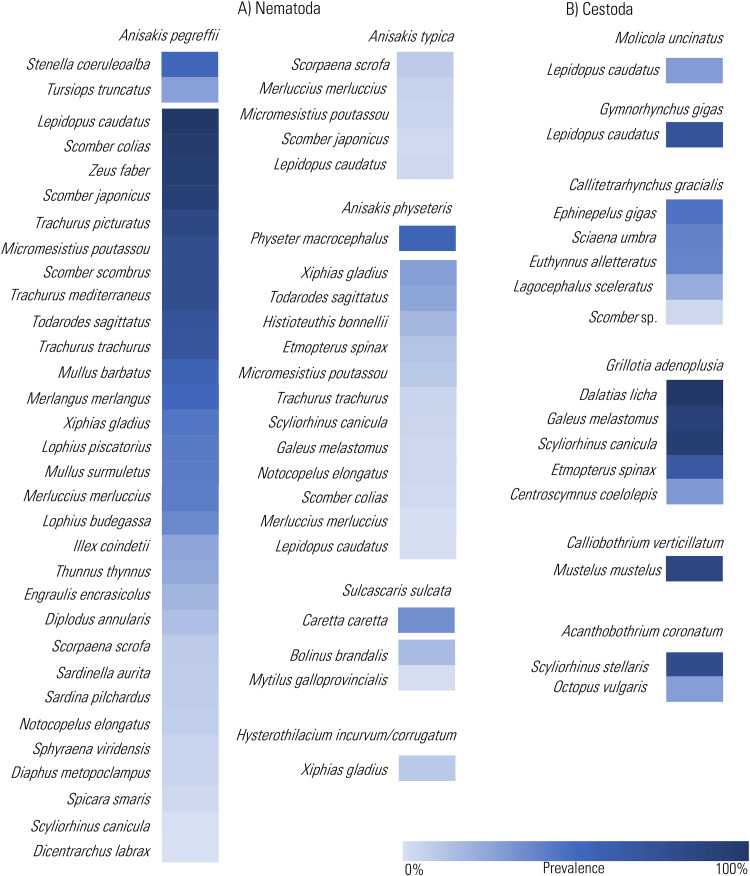
Heat Map representation of the prevalence values of the infection by the chosen TTH species among nematodes (A) and cestodes (B), so far recorded in definitive and intermediate/paratenic hosts from the Mediterranean Sea (data from cited references in Supplementary Tables 1 and 2 and through the text). The prevalence values are presented in decreasing order.

In light of these considerations, significant scientific works have been generated on these TTHs, which are the central focus of this review. Thus, we used the bulk of published information on chosen key parasites to outline their current distribution (Fig. 1), life cycle (Fig. 2) and infection values (Fig. 3) in the Mediterranean basin, and provide examples concerning their possible use as ecological indicators of the Mediterranean marine ecosystem.

### Chosen TTHs among nematodes: biodiversity and ecology

The predominant groups among nematodes found at adult stage in large organisms of the marine Mediterranean mid-upper food webs are species of ascaridoid nematodes belonging to the genera *Anisakis*, *Sulcascaris* and *Hysterothylacium.* A parasite-host list is reported in the supplementary Table 1. These genera include species having a complex life cycle, involving various marine organisms at different levels of the pelagic and/or benthic trophic webs (Fig. 2A and B). Their life cycle encompasses 4 larval stages before they reach the adult form (Klimpel and Palm, 2011) (Fig. 2A). Adults of *Anisakis* species live in the gastric chambers of marine mammals, mainly cetaceans; *Sulcascaris* species are found in sea turtles; while *Hysterothylacium* species lodge in the stomach of large teleostean fish. Female worms release fertilized unembryonated eggs into the seawater through the stools of their definitive hosts. They embryonate in seawater, maturing within the egg. The larvae moult once or twice and emerge as ensheathed, free-swimming form (see Gomes *et al.*, 2023). After a so far poorly defined free-living stage, ascaridoid larvae of these genera continue their life cycle when ingested by the first intermediate hosts. These are mostly small crustaceans, including krill for *Anisakis* spp. (Køie *et al.*, 1993, 2001). Euphausiid species such as the North Pacific krill *Euphausia pacifica*, *E. similis*, *Nyctiophanes couchii* and the Arctic krill *Thysanoessa raschii* have been found infected by *Anisakis simplex* (s.s.) (Rudolphi, 1809) (see Nascetti *et al.*, 1986) and *A. pegreffii* along the Spanish Atlantic coast (Gregori *et al.*, 2015; Cresson *et al.*, 2023). Pelagic and benthic crustaceans such as harpacticoid copepods, amphipods, isopods and mysids have been reported as intermediate hosts of *Hysterothylacium* from the Atlantic and Pacific Oceans (Køie, 1993; Klimpel and Rückert, 2005). However, knowledge concerning the first intermediate hosts of *Anisakis* and *Hysterothylacium* in Mediterranean waters is currently lacking. For *Sulcascaris*, bivalve molluscs and gasteropods serve as intermediate hosts in Mediterranean (Marcer *et al.*, 2020; Santoro *et al.*, 2020*a*, 2022*a*). In particular, the Mediterranean scallop *Pecten jacobeus* and the queen scallop *Aequipecten opercularis* were recognized as intermediate hosts in the Adriatic Sea (Marcer *et al.*, 2020). Similarly, the Mediterranean mussel *Mytilus galloprovincialis* and the purple dye murex *Bolinus brandaris* were found to serve as intermediate hosts in the Tyrrhenian Sea (Santoro *et al.*, 2020*a*, 2022*a*) (Supplementary Table 1; Fig. 2A). These host species are commonly found as part of the diet of loggerhead sea turtle *Caretta caretta* in these regions (Lazar *et al.*, 2010; Santoro *et al.*, 2019).

In the case of *Anisakis* and *Hysterothylacium*, the first intermediate hosts harbouring the third stage larvae (L3) are then eaten by various paratenic hosts, such as pelagic, demersal, benthic fish and cephalopods (Mattiucci *et al.*, 2018*b*; Tedesco *et al.*, 2018) (Supplementary Table 1; Fig. 2A and B). Other marine organisms, such as ctenophores, chaetognaths, polychaetes and ophiuroids, can also become infected by ingesting infected crustaceans and act as second intermediate or transport hosts for *Hysterothylacium* (Køie, 1993). When L3 are ingested by paratenic hosts, they can either remain encapsulated outside the visceral cavity, or migrate to the host muscle tissue in the case of *A. pegreffii*, *A. simplex* (s.s.) and *Anisakis typica* (s.l.) (Diesing, 1860) (e.g. Mattiucci *et al.*, 2018*b*; Levsen *et al.*, 2018*a*; Cipriani *et al.*, 2022*a*). In contrast, there is evidence that the L3 stages of *Hysterothylacium* and some anisakid species (i.e. *Anisakis physeteris* Baylis, 1923) do not commonly migrate into the host's muscle tissue. *Anisakis* spp. and *Hysterothylacium* spp. at L3 stage occur in several Mediterranean pelagic and mesopelagic fish and squid species (Supplementary Table 1; Fig. 2A). Co-infections with L3 *Anisakis* and *Hysterothylacium* in the same fish species in Mediterranean waters have been frequently reported (see Supplementary Table 1).

Fish and squid hosts trophically pass the parasite to their definitive hosts at the higher levels of the pelagic/benthic food webs. Once within suitable definitive hosts, namely cetaceans for *Anisakis* spp. and teleostean fish for *Hysterothylacium* spp., the L3 moults to L4 and then matures into the adult form of the parasite.

Currently, Mediterranean waters harbour at least 12 cetacean species (Notarbartolo di Sciara, 2016). The *Anisakis* species so far detected in stranded cetaceans along the coasts of the Mediterranean Sea are *A. pegreffii*, *A. simplex* (s.s.), *A. ziphidarum* Paggi *et al.*, 1988, *A. typica* sp. A and *A. physeteris* (Supplementary Table 1).

The TTH *A. pegreffii* meets the criteria for a potential ‘ecological indicator’ of anthropogenic changes in the Mediterranean Sea. Initially described at the adult stage in the Mediterranean monk seal *Monachus monachus* by Campana-Rouget and Biocca, 1955, it has not been subsequently detected in this host species. However, it is common among Mediterranean populations of cetaceans in the Delphinidae family (Supplementary Table 1). Mature female and male *A. pegreffii* have been frequently genetically identified in various delphinid species, such as the striped dolphin *Stenella coeruleoalba*, the bottlenose dolphin *Tursiops truncatus* and the Risso's dolphin *Grampus griseus*, stranded in different localities across the Central and South Mediterranean Sea (Blažeković*et al.*, 2015; Mattiucci *et al.*, 2018*b*; Cipriani *et al.*, 2022*b*). A few immature *A. pegreffii* have been genetically identified in a dwarf sperm whale *Kogia sima*, off the Tyrrhenian Sea coast (Santoro *et al.*, 2018*a*), as well as, in the Cuvier's beaked whale *Ziphius cavirostris* from the Aegean Sea (Cipriani *et al.*, 2022*b*). In addition, few adults were found in the sperm whale *Physeter macrocephalus* (Mattiucci *et al.*, 1986) (Supplementary Table 1). These findings support the hypothesis that *A. pegreffii* exhibits host preference and thus an evolutionary adaptation, within ‘oceanic dolphins’ rather than for other cetacean families, as the result of host–parasite co-speciation events (Mattiucci and Nascetti, 2008).

*Anisakis physeteris* is another chosen taxon in this review. Over the past decade, *A. physeteris* has consistently been found in stranded sperm whales along the Tyrrhenian and Ionian coasts (Mattiucci *et al.*, 1986; Cipriani *et al.*, 2022*b*), the Adriatic Croatian coast (Blažeković *et al.*, 2015) and the Aegean Sea (Cipriani *et al.*, 2022*b*) (Supplementary Table 1). Additionally, massive infections by this parasite species were noted during 2 stranding events of sperm whales in 2009 and 2013 along the southeastern coast of Italy (Mazzariol *et al.*, 2018). In the 2009 event, only 2 of 9 stranded sperm whales along the Apulia coast were found infected by a large amount (several hundreds of worms) of *A. physeteris*. In the subsequent event on the Adratic Sea coast, all 3 stranded sperm whales were infected with over 600 specimens of the parasite (Mazzariol *et al.*, 2018; Cipriani *et al.*, 2022*b*). Adult worms of *A. physeteris* were also identified in an individual of a dwarf sperm whale stranded along the Central Tyrrhenian Sea coast (Santoro *et al.*, 2018*a*). A host preference for odontocetes belonging to the Physeteridae family is evident for *A. physeteris*, which could be attributed to host–parasite co-evolutionary aspects and host feeding ecology (Mattiucci and Nascetti, 2008). *Anisakis physeteris* has been sporadically found as L3 in fish species, at low infection rates. Large, deep-dwelling cephalopods might act as reservoir hosts for this anisakid species, playing a crucial role in completing its life cycle, maintaining its distribution and infection levels. Indeed, a high parasitic load of the L3 stage of *A. physeteris* has been genetically recognized in deep sea squids of the Mediterranean Sea belonging to the family Histioteuthiidae (Palomba *et al.*, 2021*a*). These squids are preferred prey items of sperm whales. This finding, along with rare records of L3 of *A. physeteris* in pelagic and demersal fish species, suggests that *A. physeteris* has a life cycle typical of bathypelagic waters (Mattiucci *et al.*, 2018*b*) (Supplementary Table 1). Supporting this hypothesis, stranded individuals of sperm whales in 2013 had stomachs filled with beaks of the simply angel squid *Ancistroteuthis lichtensteinii*, the jumbo squid *Dosidicus gigas* and the umbrella squid *Histioteuthis bonnellii* (Mattiucci and Garibaldi, personal observation). Similarly, a dwarf sperm whale stranded on Tyrrhenian Sea coast with a high parasitic burden of *A. physeteris* had beaks of the umbrella squid and the reverse jewel squid *H. reversa* (Santoro *et al.*, 2018*a*). Likewise, a stranded sperm whale from Greece infected by *A. physeteris* contained up to 30 000 beaks of 12 squid species known to occur in the Mediterranean Sea (Rendell and Frantzis, 2016).

An additional anisakid taxon that appears to meet the aforementioned indicator criteria is *A. typica* (s.l.). Initially genetically characterized in Central Atlantic waters (Brazilian coast) and from Indian Ocean (Mattiucci *et al.*, 2002), this morphospecies has recently been recognized as a complex of sibling species, as inferred from genetic and molecular markers (see Cipriani *et al.*, 2022*a*). In fact, 2 distinct sister species, provisionally designated as *A. typica* sp. A and *A. typica* sp. B, were genetically distinguished (Cipriani *et al.*, 2022*a*). Both species were sympatrically identified at the larval stage in fish species from the Indian Ocean. This complex of species is distributed in warmer temperate and tropical waters of the world, spanning a geographical range from 35–40°N to 36°S. According to the updated finding, it appears that only 1 sibling species, i.e. *A. typica* sp. A, is currently present in the Mediterranean Sea. So far, it has been found to occur in warmer Mediterranean waters, such as off the far southeastern coast of Crypus, Crete and the Aegean Sea (Mattiucci *et al.*, 2004, 2018*a*, 2018*b*) and from the Tunisian coast (Farjallah *et al.*, 2008). This species has been found at the adult stage in a striped dolphin from Cyprus (Mattiucci and Nascetti, 2008) and at larval stage in fish hosts of pelagic and mesopelagic domain (Mattiucci *et al.*, 2004, 2018*b*; Cipriani *et al.*, 2018*a*) (Supplementary Table 1; Fig. 3).

Concerning the occurrence of other species of *Anisakis* in Mediterranean Sea, records of *A. ziphidarum* are sporadic (see Mattiucci *et al.*, 2018*b*). Its geographical range is mainly linked to the distribution of its definitive hosts, i.e. the beaked whales (see Mattiucci *et al.*, 2018*b*). Its first record in the Mediterranean Sea was in an individual of the Cuvier's beaked whale off the Ionian coast (Paggi *et al.*, 1998) (Supplementary Table 1). It is rarely reported, at L3 stage, in pelagic and demersal fish species, worldwide (see Mattiucci *et al.*, 2018*b*). Instead, in the Mediterranean basin, the species was genetically recognized in the deep-dwelling myctophid meso-benthopelagic spothead lantern fish *Diaphus metopoclampus* caught in the eastern Ionian Sea (Gaglio *et al.*, 2018) (Supplementary Table 1). Habitat modelling for populations of the Cuvier's beaked whale in the Mediterranean Sea (Podestà *et al.*, 2016) confirms its preference for deep waters (>200 m). These findings suggest that the life cycle of *A. ziphidarum* involves deep sea species, such as deep-sea squids. Its scarcity in Mediterranean records (Supplementary Table 1) can be attributed to the rare populations of beaked whales, the suitable definitive hosts of *A. ziphidarum* (Mattiucci and Nascetti, 2008) so far recorded in the region (IUCN, 2020), as well as to the limited parasitological studies so far focused on deep sea species. Therefore, this TTH parasite was not chosen as possible sentinel parasite in this review.

*Anisakis simplex* (s.s.) has an Arctic-Boreal distribution (see Mattiucci *et al.*, 2018*b*), and has been rarely documented in Mediterranean waters. Here, *A. simplex* (s.s.) has been occasionally detected at the larval stage in several fish species in the Alboran Sea, the westernmost portion of the Mediterranean Sea (see Mattiucci *et al.*, 2018*b*; Molina-Fernández *et al.*, 2018; Roca-Geronès *et al.*, 2021), which is considered as part of the Atlantic waters than the Mediterranean Sea (Hidalgo *et al.*, 2018). Spot occurrence of *A. simplex* (s.s.) has been also documented in the eastern part of the Mediterranean Sea. The presence of this species in the basin is likely linked to the migratory routes of the intermediate/definitive hosts transporting the parasite, such as the bluefin tuna *Thunnus thynnus* and the striped dolphin (Mladineo and Poljak, 2014; Blažeković *et al.*, 2015). Adult specimens of *A. simplex* (s.s.) are extremely rare in the Mediterranean. This is likely due to the Mediterranean Sea temperature as a limiting abiotic factor for the eggs hatching and first stage larval survival of the parasite (Gomes *et al.*, 2023). Only 1 adult, found in co-infection with several specimens of *A. pegreffii*, was recorded in a striped dolphin from off Sicilian coast (Cavallero *et al.*, 2014) (Supplementary Table 1).

Among the endoparasites of cold-blooded marine animals, the anisakid *Sulcascaris sulcata* (Rudolphi, 1819) Hartwich, 1957 has been chosen in this review. Adults of *Sulcascaris* have been described in the gastric lumen of 3 marine turtle species: the loggerhead sea turtle, the green turtle *Chelonia mydas* and Kemp's ridley sea turtle *Lepidochelys kempii* in Atlantic and Pacific waters (Berry and Cannon, 1981). In the Mediterranean Sea, 3 sea turtle species (i.e. the leatherback turtle *Dermochelys coriacea*, the green turtle and the loggerhead turtle) have been recorded (Casale and Margaritoulis, 2010). From this basin, *S. sulcata* has been reported in loggerhead sea turtles from the Adriatic (Manfredi *et al.*, 1998; Gračan *et al.*, 2012; Santoro *et al.*, 2019; Marangi *et al.*, 2020; Gentile *et al.*, 2021) and Tyrrhenian Sea (Santoro *et al.*, 2019), as well as from North African coastline (Sey, 1977), with prevalence varying from 18 to 33% (Supplementary Table 1; Fig. 3).

Worms of the genus *Hysterothylacium* have been mostly recorded at adult stage in the stomachs of large teleostean predators inhabiting the Mediterranean waters. Within the genus, *Hysterothylacium incurvum* (Rudolphi, 1819) Deardorff and Overstreet, 1980 and *H. corrugatum* (s.l.) Deardorff and Overstreet, 1981 are dominant and specialized nematodes of the swordfish, having this fish as their definitive host (Supplementary Table 1; Fig. 2B). Their occurrence in swordfish has been documented both in the Mediterranean (Mattiucci *et al.*, 2014, 2015*a*, 2015*b*) as well from other geographical areas included in the host range, such as the North-East and equatorial-tropical Atlantic waters (Garcia *et al.*, 2008, 2010). However, no data are currently available for the larval occurrence of these species in intermediate/paratenic hosts. The presence of numerous squid beaks in swordfish stomachs examined during parasitological surveys leads to the hypothesis that the L3 might occur in deep-sea squid species that have not yet been investigated (Garcia *et al.*, 2008; Mattiucci *et al.*, 2015*a*).

Regarding the occurrence of other *Hysterothylacium* species, there is only limited evidence of adults in other large teleostean species (Table 1) (Ortis and Paggi, 2008). However, combined parasitological surveys and molecular analyses have identified L3 of *H. aduncum* (s.l.) in pelagic and demersal fish from Mediterranean waters (Supplementary Table 1). *Hysterothylacium aduncum* is currently the most common species, found at larval stage in more than 40 fish hosts (Petter and Radujkovic, 1989) (Supplementary Table 1). Larval stages of *Hysterothylacium* spp. have also been detected in various cephalopods, particularly oegopsid squids (Tedesco *et al.*, 2020*a*, and references therein). Despite the abundance of larval stage reports, data on the infection by *H. aduncum* at the adult stage from the Mediterranean Sea are missing (Supplementary Table 1). Whereas this parasite species has been constantly recorded at adult stage in gadoid fish from NE Atlantic waters (see Levsen *et al.*, 2022 and references therein). Consequently, despite its large occurrence at larval stage in pelagic and demersal Mediterranean food webs, *H. aduncum* was not chosen as possible sentinel parasite in this review, for the lack of consistent data on its definitive hosts.

**Table 1.** Chosen TTHs as sentinels of food-webs stability, environmental pollution and sea temperature change in the Mediterranean Sea

	Metrics	Food-webs stability		Environmental pollution	Sea temperature change
		Infection levels and relative proportions	Genetic variability	Estimates of pollutants	Relative proportions
Nematoda					
*Anisakis pegreffii*		+ +	+ +	±	+
*Anisakis typica*		+ +	+	±	+ +
*Anisakis physeteris*		+ +	+ +	±	nd
*Sulcascaris sulcata*		+ +	+ +	±	+
*Hysterothylacium incurvum*		+ +	nd	nd	nd
*Hysterothylacium corrugatum*		+ +	nd	nd	nd
Cestoda					
*Gymnorhynchus gigas*		+ +	nd	+ +	nd
*Molicola uncinatus*		+ +	nd	+ +	nd
*Grillotia adenoplusia*		+ +	+ +	+ +	+
*Callitetrarhynchus gracilis*		+ +	nd	+ +	nd
*Calliobothrium verticillatum*		+ +	nd	+ +	nd
*Acanthobothrium coronatum*		+ +	+	+ +	nd

Metrics: measurable characteristics of sentinel parasites as tools for ecosystem monitoring. A good ( + +), moderate ( + ) or low ( + /–) potential level of contribution of the parasite species to a specific change, according to the currently assessed data. nd: not enough data.

### Chosen TTHs among cestodes: biodiversity and ecology

Current knowledge on the species diversity of cestodes maturing in mid-upper trophic webs in the Mediterranean environment primarily concerns those from elasmobranchs. However, it is characterized by a mix of old and limited historical records. A parasite-host list is reported in the supplementary Table 2. Most of the observations report the parasite occurrence in intermediate/paratenic teleostean fish. Recent investigations, employing an integrative taxonomy approach, are consistently revealing a wide distribution and diversity of these cestodes in the Mediterranean. In particular, the trypanorhynch tapeworms stand out as the most extensively reported. Other groups such as Tetraphyllidea, Oncoproteocephalidea, Rhinebothriidea and Phyllobothriidea have historical records, but have received relatively limited recent attention (Supplementary Table 2). Indeed, according to historical data, the Mediterranean Sea seems to represent a hotspot of diversity for trypanorhynchs, harbouring around the 14% of the currently recognized species worldwide. The most represented families (by number of species) belong to Lacistorhynchidae (14 species), Eutetrarhynchidae (12 species), Tentaculariidae (5 species), Gymnorhynchidae (4 species) and Sphyriocephalidae (4 species) (Supplementary Table 2). These families account for about 85% of all trypanorhynch species found to date in the Mediterranean.

In general, trypanorhynchs are characterized by a complex life cycle (Fig. 2C and D), involving various marine organisms across different marine trophic levels. It has been suggested that different species have evolved different life-cycle strategies, occupying different ecological niches, also in terms of vertical distribution (Palm, 2004). It is noteworthy that many of them exhibit a strong preference for a particular definitive host (Palm and Caira, 2008), following the most common feeding ecology and depth range of this host. In general, their life cycle stages typically consist of a procercoid, followed by a plerocercus, or in some species, a plerocercoid or merocercoid, and ultimately the adult stage. Two hypothetical life cycle patterns have been proposed (Palm, 2004). In the first scenario, the life cycle encompasses 3 hosts, with copepods acting as the first host and invertebrates as the second intermediate host, as observed in the Eutetrarhynchidae (Palm, 2004). Alternatively, copepods can be the first host, and pelagic euphausiids can be the second intermediate host, as observed in Gymnorhynchidae. In the second pattern, the life cycle might involve 3 hosts, with copepods as first hosts and fish as obligatory second intermediate hosts, as is the case for many of the Lacistorhynchidae. A life cycle fully elucidated in nature is that of *Grillotia erinaceus* (van Beneden, 1858) Guiart, 1927 (Lacistorhynchidae). Operculate eggs released in sea water shed free-swimming coracidia in 5 days. The copepods *Acartia longiremis* or *Pseudocalanus elongates* serve as first intermediate hosts. The plerocercus develops in fish (especially in Gadiformes) while *Raja* spp. serve as definitive hosts (Palm, 2004). The life cycles of Lacistorhynchidae can also extend to 4 hosts, where copepods are the first host, followed by schooling fish and large predatory fish as the second and third intermediate hosts, respectively. For example, among the Lacistorhynchidae, *Callitetrarhynchus gracilis* (Rudolphi, 1819) Pintner, 1931 exhibits a 4-host trophic chain life cycle. The plerocercus with small form generally occurs in smaller fish species, as clupeids, while the large form is present in larger fish, as scombrids, paralichthyids and balistids (Palm, 2004).

This review has focused on 4 trypanorhynch species. They were chosen due to the new insights gained about their occurrence in intermediate/paratenic hosts, and on occasion, in definitive hosts of marine organisms inhabiting the Mediterranean Sea. The chosen species are: *Molicola uncinatus* (Linton, 1924) Palm, 2004 and *G. gigas*, belonging to the family Gymnorhynchidae; *Grillotia adenoplusia* (Pintner, 1903) Palm, 2004, and *C. gracilis*, which are representatives of the family Lacistorhynchidae.

*Molicola uncinatus* has been documented across various global regions. Notably, the larval stages have been found in scombriformid fish from diverse locations, including Cape Campbell (Cook Strait, New Zealand), the South African coast and Mediterranean Sea (see Palm, 2004; Palomba *et al.*, 2021*b*; Santoro *et al.*, 2022*b*). However, as a sporadic finding, it was also reported in Tetraodontiformes and Carangiformes (Palm, 2004). More specifically, in the Mediterranean Sea, this species has been genetically identified along the coast of Malta, in the silver scabbardfish *Lepidopus caudatus* (Palomba *et al.*, 2021*b*) and in the Atlantic pomfret *Brama brama* from the Tyrrhenian and Ionian Seas (Santoro *et al.*, 2022*b*). The high parasitic burden (*A* ≈ 28.8) of *M. uncinatus* within the silver scabbardfish strongly indicates the successful functioning of the parasite's life cycle in the Mediterranean (Fig. 3). The definitive host for the species is the common thresher *Alopias vulpinus* (Palm and Caira, 2008). Even if *M. uncinatus* seems to show a strong species-specific association with this definitive host, however, no infection data about this cestode are so far available from stranded common thresher in the Mediterranean Sea. The population of the common thresher, found between surface waters and depths of 360 m in the Mediterranean Sea, is currently classified as critically endangered (IUCN). Also in the Mediterranean Sea, another significantly prevalent species is *G. gigas*. Here, it has been observed, at adult stage, in members of the Lamnidae, i.e. the great white shark *Carcharodon carcharias*, the porbeagle shark *Lamna nasus* and also the angular roughshark *Oxynotus centrina* of the Oxynotidae.

Larval *G. gigas* have been observed in the Atlantic pomfret, with high levels of infection from both Tyrrhenian and Ionian Seas, and the silver scabbardfish captured in the Central Mediterranean Sea (Fig. 3) (Manfredi *et al.*, 1993; Giarratana *et al.*, 2014; Palomba *et al.*, 2021*b*; Santoro *et al.*, 2022*b*).

The larvae of *M. uncinatus* and *G. gigas* may coexist in sympatry and syntopy. However, identifying Gymnorhynchidae larvae based solely on morphological criteria can be a challenging or sometimes impossibile, explecially when their tentacles are partially or completly invaginated, hindering the examination of crucial taxonomic features. In such cases, the use of molecular analysis becomes essential for precise species identification. In the Mediterranean basin, another species recently reported is *Gymnorhynchus isuri* Robinson, 1959 found in the sunfish *Mola mola* (Santoro *et al.*, 2020*b*). Originally documented in the north, southwestern and northeast Atlantic, as well as the Tasman Sea in the southwestern Pacific, *G. isuri* is known to infect the shortfin mako *Isurus oxyrinchus* and the blue shark *Prionace glauca* (Palm *et al.*, 2009; Penadés-Suay *et al.*, 2022). The occurrence of this species in Mediterranean waters is probably connected to the migratory pathways of sunfish (Santoro *et al.*, 2020*b*).

Seven species of the genus *Grillotia* belonging to the family Lacistorynchidae have been mainly recorded in the Mediterranean basin (Beveridge and Campbell, 2013; Dallarés, 2016) (Supplementary Table 2). Among these, *G. heptanchi* (Vaullegeard, 1899) Dollfus, 1942 and *G. adenoplusia* have been observed as adults in sharks, while *G. erinaceus* has been found as adults in skates and rays (Beveridge and Campbell, 2013). Until a decade ago, only limited literature was available on *Grillotia* infections in the Mediterranean, with only *G. erinaceus* having undergone molecular identification. However, because of its sporadic and old records in Mediterranean region, *G. erinaceus* has not been considered in this review. Recent advancements in molecular systematics have expanded knowledge of *Grillotia* species in the Mediterranean Sea. Plerocerci of *Grillotia* were detected in the angler fish *Lophius piscatorius* (Santoro *et al.*, 2018*b*) and in deep-dwelling sharks (Dallarés, 2016; Santoro *et al.*, 2021*b*). Morphological and molecular studies, recently performed on these plerocerci, recognized them as belonging to the species *G. adenoplusia* (Isbert *et al.*, 2023). Specifically, plerocerci of this cestode have been documented in the blackmouth catshark *Galeus melastomus*, the Portuguese dogfish *Centroscymnus coelolepis* and the velvet belly lanternshark *Etmopterus spinax* from the Western Mediterranean coast of Spain (Dallarés, 2016; Dallarés *et al.*, 2017*a*, 2017*b*; Isbert *et al.*, 2023). No reports of *Grillotia* specimens were detected in the small-spotted catshark *Scyliorhinus canicula* from the same area. The prevalence of infection ranged from 17 to 100%, with a maximum abundance value of 84.6 recorded in the blackmouth catshark (Fig. 3).

Likewise, in the Tyrrhenian Sea, the velvet belly lanternshark, the blackmouth catshark and the small-spotted catshark have also been found to host these plerocerci (Santoro *et al.*, 2021*b*). In these cases, prevalence ranged from 82.0 to 100% with an abundance of 181.65 in the blackmouth catshark(Santoro *et al.*, 2021*a*) (Fig. 3). The presence of this TTH has been associated with the consumption of myctophid fish, which are known to prey on copepods, suggesting that these could potentially serve as intermediate hosts for *G. adenoplusia* (Dallarés, 2016; Santoro *et al.*, 2022*c*). The bluntnose sixgill shark *Hexanchus griseus* was established as the definitive host for *G. adenoplusia* (Beveridge and Campbell, 2013).

In this review, *C. gracilis*, a member of the Lacistorhynchidae family, has also been considered. According to Palm (2004), *C. gracilis* has a trophically linked 4-host life cycle. In the Eastern Mediterranean Sea, larger fish species such as the bullet tuna *Auxis rochei*, the little tunny *Euthynnus alletteratus*, greater amberjack *Seriola dumerili* and the bluefin tuna act as hosts for the plerocerci of *C. gracilis* (Abdelsalam *et al.*, 2016; Gabel *et al.*, 2022; Morsy *et al.*, 2022). High infection levels by this parasite have been reported in these hosts. For instance, the species infects the little tunny off the coast of Alexandria with a prevalence of 38.7% (Abdelsalam *et al.*, 2016). Notably, the parasite has also been identified in the little tunny populations in Turkey, where the prevalence soars to 91.3% (Akmirza, 2006) (Fig. 3). Adults of *C. gracilis* were found in carcharhinid sharks (*Carcharhinus* spp.), although these have yet to be identified in the Mediterranean Sea.

The Tetraphyllidea, from which 3 new orders have recently emerged, namely Rhinebothriidea, Onchoproteocephalidea and Phyllobothriidea (Caira and Jensen, 2014), has traditionally been considered as one of the most speciose orders, worldwide. To date, reports of those cestodes infecting Mediterranean elasmobranchs are mostly based on morphological data, with few exceptions (Supplementary Table 2). The most recently studied species in the Mediterranean Sea are the tetraphyllidean *Calliobothrium verticillatum* (Rudolphi, 1819) (Fig. 2E) and the onchoproteocephalid *Acanthobothrium coronatum* (Rudolphi, 1819) Blanchard, 1848 (Fig. 2F) (Tedesco *et al.*, 2020*b*, 2020*c*; Santoro *et al.*, 2021*b*).

Originally described from an unspecified shark species of Italy, *C. verticillatum*, the most representative species of its genus, has since been documented in the common smooth-hound *Mustelus mustelus* from the Northeastern Atlantic. Later, Euzet (1959) reported it infecting the Mediterranean common smooth-hound. Tedesco *et al.* (2020*b*) recently confirmed the occurrence of this host–parasite relationship in Mediterranean waters, specifically in the Adriatic Sea. Given these reports, the species demonstrates a high level of specificity with respect to its definitive host species. Indeed, *C. verticillatum* matures in the spiral valve of common smooth-hound. In this host, it produces hexacanth embryos that are released from gravid proglottids. The next stage involves the decapod grey hermit crab *Pagurus pollicaris* as first intermediate host, which ingests hexacanths. Inside the body of the hermit crab, the embryo undergoes development, first into a procercoid and then into a plerocercoid larva (see Tedesco *et al.*, 2020*b*) (Fig. 2E). Currently, no information is available on intermediate hosts for this cestode species in the Mediterranean Sea.

Knowledge regarding species of the genus *Acanthobotrium* in the Mediterranean are generally outdated, with few exceptions, such as in the case of *A. coronatum.* Previously known for its non-specific associations with elasmobranch hosts, *A. coronatum* has recently been discovered to exclusively thrive in the nursehound *Scyliorhinus stellaris* from the Tyrrhenian Sea (Santoro *et al.*, 2022*d*). This knowledge has been recently acquired from a parasitological investigation which thoroughly explored a diverse array of potential host species (Santoro *et al.*, 2022*d*). Generally, members of the onchoproteocephalidean family, to which *A. coronatum* belongs, present a 3–5 host life cycle (Fig. 2F). This encompasses copepods as first, molluscs as second intermediate hosts, while fish act as intermediate or paratenic hosts (Jensen and Bullard, 2010). A comparative molecular analysis, including sequences of *A. coronatum* from the nursehound and sequences of an unidentified *Acanthobothrium* collected from the common octopus *Octopus vulgaris*, has identified this cephalopod as a suitable intermediate host of *A. coronatum* (Tedesco *et al.*, 2020*c*; Santoro *et al.*, 2022*d*). In support of this, the common octopus has been confirmed as primary prey item of the nursehound (Santoro *et al.*, 2022*d*).

## TTHs as ‘sentinels’ of anthropogenic impacts in marine ecosystems

Marine helminth parasites with complex life cycles can be directly or indirectly affected by abiotic (i.e. sea water temperature, salinity, pollution) and biotic (i.e. host-species demography) factors. The autoecology of TTHs is the result of a long adaptation to their environment and their hosts, often including both homeothermic and heterothermic organisms. Thus, environmental changes, such as those caused by anthropogenic impacts, could significantly alter the dynamics of these organisms deeply embedded in the marine ecosystem. For this reason, TTHs interactions with the environment and their intermediate/definitive hosts constitute delicate biodiversity-functioning relationships. These relationships can be easily disrupted, either directly due to environmental changes or indirectly as a result of alterations in host dynamics (Marcogliese, 2005; Hudson *et al.*, 2006; Mattiucci and Nascetti, 2008; Palm, 2011; Byers, 2021; Selbach *et al.*, 2022) (Fig. 2). Stressors affecting host population dynamics have the potential to compromise the transmission pathways of parasites, leading to changes in parasite distribution and number, even on a regional scale.

The possible contributions of TTHs as ‘sentinels’ of anthropogenic changes are proposed in Table 1. Measurable attributes to be used as metrics in future monitoring, at both spatial and temporal scale level, include those concerning their distribution, infection levels, relative proportions and genetic diversity. Some examples of the metrics evaluated in chosen TTHs from the Mediterranean Sea are here presented.

### Infection levels of TTHs as indicators of food-web stability

Successful completion of TTHs life cycles requires suitable marine environmental conditions and stable food chains in which parasites are dependent on their hosts, to survive, develop and reproduce. Thus, the main factor shaping the distribution and infection levels of a parasite species in a local marine ecosystem is the availability of appropriate definitive and intermediate hosts, which in turn, maintains a high probability of multi-host parasite transmission (Cirtwill *et al.*, 2016). Among the drivers responsible for infection dynamics of chosen TTHs in the Mediterranean Sea, the distribution and the demography of both their definitive (i.e. cetaceans, sea turtles, elasmobranchs) and intermediate/paratenic (i.e. crustaceans, fish and molluscs) hosts play a determinant role. These factors contribute, in turn, to maintain the effective population size of a TTH species at regional level.

The infection levels of *A. pegreffii* in its paratenic fish host species (i.e. the European hake) vary significantly across fishing grounds in the western and eastern Mediterranean Sea. Indeed, in recent years, the observed infection rates with the parasite species in fish sampled from the Central and Southern Adriatic Sea were notably high (*P*≈95%; A≈128) than those recorded in the same fish host species sampled from Tyrrhenian Sea (Cipriani *et al.*, 2018*a*). Historical data indicate that the European hake, collected from the same area (i.e. Adriatic Sea) and same sized fish, did not show high infection rates by *A. pegreffii* (*P*≈73.3; A≈6.1) (Mattiucci *et al.*, 2004). It can be hypothetically considered that environmental changes could have impacted parasite infection levels in this region. Oceanographic currents, along with nutrient-rich upwelling, could support a high population of planktonic organisms, the first hosts in the life cycle of the parasite. This abundance of hosts also benefits small pelagic fish such as anchovies and sardines, which serve as paratenic hosts for the parasite in the Adriatic. Moreover, notable populations of local bottlenose dolphins, the definitive host of the parasite species, have been observed, with these dolphins heavily preying on anchovy and hake (Holcer, 2012). Substantial *A. pegreffii* infections have indeed been detected in dolphins stranded along the Croatian coast (Blažeković *et al.*, 2015). Consequently, these are optimal conditions for completion of the *A. pegreffii* life cycle, sustaining the elevated infection levels so far found in commercially important fish species (Cipriani *et al.*, 2018*a*, 2018*b*). Nonetheless, these abiotic and biotic factors and anthropogenic influence appear to be also responsible for the increased parasite infection in this Mediterranean Sea fishing ground. Indeed, an ‘anthropogenic shortcut’ in the life cycle of the parasite could occur as a consequence of the widespread practice of eviscerating fish directly onboard vessels and discarding their viscera at sea. This practice, aimed at removing viscera from heavily *Anisakis* larvae-infected fish, whose presence could compromise the value of the fish at landing, might lead to the inadvertent direct introduction of *A. pegreffii* larvae into the sea. Despite current Italian regulations suggesting proper on-land disposal, the discarded fish entrails carrying live *A. pegreffii* larvae serve as potential food for other fish and cetaceans, thereby facilitating the parasite's life cycle. As a result, dolphins following trawlers and consuming these discarded entrails and larvae, can accumulate more *A. pegreffii*. This contributes to a persistent high parasite burden of *A. pegreffii* in the Adriatic Sea's coastal fisheries, such as those so far reported (Mladineo and Poljak, 2014; Cipriani *et al.*, 2018*a*, 2018*b*).

In contrast, *A. pegreffii* has shown in the European hake, so far, lower values of prevalence and abundance in the same fish species from other Mediterranean Sea regions, for instance: from Ligurian Sea *P* ≈ 27.6, A ≈ 0.54; Central Tyrrhenian Sea *P* ≈ 44.2, A ≈ 1.39; Alboran Sea *P* ≈ 26.2, A ≈ 0.73. Interestingly, a temporal analysis from 1998 to 2019 of *A. pegreffii* infections in the European hake from the Atlantic Spanish coast (an area comprised into the parasite's geographical range) revealed an increasing trend in both prevalence and abundance of the infection (Diez *et al.*, 2022). The authors correlated this trend with a corresponding rise in stock biomass of fish species, such as the European hake, the European anchovy *Engraulis encrasicolus* and the Atlantic mackerel *Scomber scombrus*, which also serve as suitable intermediate/paratenic hosts for *A. pegreffii* (Diez *et al.*, 2022). Therefore, the higher fish host demography in Spanish Atlantic waters (Diez *et al.*, 2022) contributes to a larger parasite population size and higher levels of infection. Furthermore, the same parasite species, in its populations in Austral regions (i.e. Argentine and New Zealand waters) exhibits higher levels of prevalence and abundance (Hossen *et al.*, 2021; Irigoitia *et al.*, 2021). This suggests that the transmission route of the parasite life cycle in these extra-Mediterranean waters, included in the range of distribution of the parasite species (see Mattiucci *et al.*, 2018*b*), is maintained at a higher intensity.

This would be related to the substantial demography of its suitable definitive (Hammond *et al.*, 2013; Magera *et al.*, 2013) and intermediate/paratenic hosts in those waters, probably characterized by more stable ecosystem dynamics. Conversely, we could hypothesize that the Mediterranean pelagic and mesopelagic trophic webs, exploited by *A. pegreffii*, may be less stable and therefore unable to maintain a population size comparable to those in other regions. In support of this observation, we should consider the population status of the most common definitive hosts of *A. pegreffii* in the Mediterranean, i.e. dolphins. Despite recent conservation efforts aimed at monitoring and protecting marine mammals in Mediterranean waters (for instance, the Santuario dei Cetacei, ‘Pelagos’ in the basin area between the Ligurian and Tyrrhenian Sea), the population size of certain dolphin species is lower than in other oceanographic areas (IUCN, 2020), thus supporting smaller parasite population abundance values. Indeed, unfortunately, Mediterranean marine mammals and sea turtles are currently affected by various anthropogenic disturbances, such as fisheries by-catch and plastic pollution (IUCN, 2020). For instance, sperm whales stranded in 2009 along the Italian Ionian Sea were found uninfected by anisakid worms, and were instead loaded with a large quantity of plastic waste (Mattiucci and Marcer, personal observation). In addition, sperm whale mortalities have been reported as caused by plastic ingestion (De Stephanis *et al.*, 2013). Meanwhile, the high infection rate with *A. physeteris* found in both Mediterranean sperm whales (Mazzariol *et al.*, 2018) and dwarf sperm whales (Santoro *et al.*, 2018*a*) seems to indicate a well-established life cycle of the parasite in the Mediterranean Sea. Thus, the occurrence of *A. physeteris* could be used as an ecological indicator to monitor the stability of trophic webs. These would encompass the aforementioned physeteriids and deep-sea squid species such as histiotheuthids. These squids are not directly impacted by overfishing activities, and have a significant parasite burden (on average, A ≈ 18) (Palomba *et al.*, 2021*a*) (Fig. 3).

Infections by the anisakid *S. sulcata* in the Mediterranean Sea are strongly linked to the abundance of its definitive host, the loggerhead sea turtle and the availability of its prey. Indeed, juveniles and adults of this sea turtle species are present throughout the entire basin, concentrating their foraging activity along the North Adriatic Sea and Central Mediterranean shelf; lower frequencies are reported from the Southern Ionian and Sicilian areas (Casale *et al.*, 2020). Host migratory activity throughout the Mediterranean plays a significant role in shaping the transmission and infection levels observed with *S. sulcata* in the North Adriatic and Tyrrhenian Seas (Santoro *et al.*, 2019; Marcer *et al.*, 2020). In addition, the differential foraging habits of juvenile and adult loggerhead sea turtles, as well as their distinct habitats in these basins, are responsible for defining the distribution of *S. sulcata*. The prevalence of *S. sulcata* was found to be significantly higher in loggerhead sea turtles from the Adriatic Sea with respect to the Tyrrhenian Sea. However, while in the Adriatic Sea both early and large juveniles of the loggerhead sea turtles were found infected, in the Tyrrhenian Sea only larger individuals were parasitized. These differences seem to be the result of regional environmental habitat characteristics, and prey availability for the loggerhead sea turtle in these basins (Santoro *et al.*, 2019). Both juveniles and adult loggerhead sea turtles are known to feed on the benthic organisms in the Northern Adriatic Sea, here including bivalves and gastropods. Adults forage instead in shallower coastal waters of the Tyrrhenian Sea on benthic molluscs, such as the Mediterranean mussel (Casale *et al.*, 2008) which are intermediate hosts of *S. sulcata* (Santoro *et al.*, 2019; Marcer *et al.*, 2020). Thus, in the Adriatic Sea, the benthic trophic web seems to support a higher probability for juveniles to become infected by *S. sulcata*. The increasing trend showing high parasitic load (*A* = 100) found in adult sea turtles also suggests that the parasite accumulates in its definitive hosts (Santoro *et al.*, 2019). Thus, the abundance of the loggerhead sea turtle recently recorded in the Mediterranean (Di Matteo *et al.*, 2022) seems to play a role in sustaining the population size of its specific anisakid endoparasite.

Significant differences in the infection levels with the adult raphidascarid nematodes *H. incurvum* and *H. corrugatum* (s.l.), specific endoparasites of the swordfish, were observed between the Mediterranean Sea and Atlantic waters (Mattiucci *et al.*, 2014). These differences suggest the existence of distinct stocks of the swordfish in these waters, as also indicated by the population genetics of the fish host stocks (see Mattiucci *et al.*, 2015*a*, 2015*b*). High infection rates, in terms of both prevalence and mean intensity, were recorded for *Hysterothylacium* spp. in swordfish from the Atlantic tropical-equatorial waters. Indeed, from this area, *H. incurvum* showed, on average, prevalence, P≈ 90% and mean intensity, Im ≈ 39.7; while the species *H. corrugatum* (s.l.) reached, on average, the value of *P* ≈ 87.3%, and Im ≈ 20. Similarly, in the Atlantic Iberian swordfish population, *H. incurvum* showed *P* = 100% and, on average Im ≈ 18.6, while *H. corrugatum* (s.l.) reached the values of *P* = 100% and on average Im ≈ 25.7 (Garcia *et al.*, 2010). Conversely, lower levels of infection were observed, over the same temporal but at different spatial scale, in the Mediterranean stock: *H. incurvum*: *P* ≈ 14.3, on average, Im ≈ 5.3; *H. corrugatum* (s.l.): *P* ≈ 14%, on average Im ≈ 5.8 (Mattiucci *et al.*, 2014). These differences in the parasitic load of *Hysterothylacium* species in the swordfish populations could be attributed to variations in the foraging patterns of the swordfish in different areas. This variation is likely linked to the availability of food items, mostly deep-sea squid species, which serve as suitable intermediate hosts for these species of *Hysterothylacium*. The presence of squid beaks in the gastric contents of swordfish from tropical-equatorial waters heavily infected by these *Hysterothylacium* species further supports this hypothesis (Garcia *et al.*, 2011) (Fig. 3). Thus, the composition and parasitic burden with *Hysterothylacium* spp. in swordfish stocks from different fishing grounds are likely the outcome of a combination of factors, including the population size of their swordfish definitive host as well as the availability and demography of its favourite prey items. In accordance, while the North Atlantic swordfish population benefited from management actions that have rebuilt its stock (Neilson *et al.*, 2013), the Mediterranean swordfish stocks are still under management to avoid a possible stock collapse in the next generations (Tserpes *et al.*, 2011). Thus, within the framework of a holistic approach in fisheries management, the parallel monitoring of infection levels with these TTHs would provide an indication of habitat or stock deterioration of the Mediterranean swordfish.

Concerning cestodes, in the Mediterranean Sea, specifically within the Gulf of Naples, a notable prevalence and abundance (*P* ≈ 53.6; abundance, A ≈ 5.32) of the cestode *A. coronatum* has been recently documented (Santoro *et al.*, 2021*b*, 2022*d*). This parasite species has the nursehound shark as its suitable definitive host. This elasmobranch species is demersal, primarily dwelling along the continental shelves and slopes. The nursehound shark's diet predominantly comprises a variety of benthic organisms, with cephalopods often as a substantial dietary component (Santoro *et al.*, 2022*d*). The nursehound shark's dietary preferences and its ecology are significant factors influencing the parasite's transmission route. Thus, the high prevalence of *A. coronatum* in the Central Tyrrhenian area, off the Gulf of Naples seems to indicate a stable population size of its definitive host in this geographical area. Supporting this, the Santa Croce Bank in the Gulf of Naples is recognized as the main known Mediterranean breeding ground for the nursehound shark (de Sabata and Clò, 2013). A similar trend of infection levels (*P* = 85%, *A* = 7.3) by *C. verticillatum* has been documented in the common smooth-hounds captured in the Tyrrhenian Sea (our unpublished data) (Fig. 3). This could suggest the presence of a stable coastal trophic web, with the species-specific common smooth-hound as the definitive host and the pagurus as intermediate one.

### Intraspecific genetic variability of TTHs as an indirect indicator of food web stability

The demographic dynamics of host populations and the environmental abiotic factors also have a role in shaping the genetic structure and diversity of nematode and cestode populations (Mattiucci and Nascetti, 2008; Isbert *et al.*, 2023).

For instance, it has been postulated that multi-host parasites with life cycles completed in both poikilothermic and homeothermic hosts (for instance, *Anisakis* spp.) exhibit higher levels of genetic polymorphism and heterozygosity compared with single-homeothermic host species (Bullini *et al.*, 1986). Such intraspecific genetic variation is fundamental for the species adaptive capacity to changing environments (May, 1994). In the case of multi-host parasites, it was indeed hypothesized that within the gene pool of a parasite species, a given allele may confer greater fitness in one stage of the life cycle, while another allele may function optimally in another group of hosts of its life cycle (Bullini *et al.*, 1986). In addition, estimates of genetic variability can shed light on the effect of ecological traits and bottleneck events that may have occurred historically within the gene pools of multi-host helminths, potentially as a result of anthropogenic-driven environmental changes (Mattiucci and Nascetti, 2008). Indeed, assessment of genetic variability parameters – i.e. the estimates of genetic polymorphism, rare alleles and expected heterozygosity (*He)* – of a parasite species from an oceanographic area can assist with evaluation of the parasite population status, and provide an indicator of stable or changing environmental conditions. Any habitat perturbation directly disrupting the abiotic conditions for egg hatching, or, through a ‘domino effect’, any habitat perturbation that decreases host numbers could negatively impact local parasite populations. Consequently, this sequence of events increases the probability of genetic erosion within the parasite population gene pool resulting in the loss of general polymorphism due to genetic drift phenomena and loss of rare alleles (Mattiucci and Nascetti, 2008; Pauls *et al.*, 2013; Almeida-Rocha *et al.*, 2020). In agreement with this hypothesis, a significant positive correlation between the genetic variability values of a parasite population and its abundance (as a proxy of population size of the parasite species) of TTHs parasites and their hosts has been documented (Zarlenga *et al.*, 2014). An attempt to positively correlate genetic variability values with the effective population size (*Ne*) of a parasite species as a conservation measure has been also recently speculated for species of nematomorphs (De Vivo *et al.*, 2023). A research on anisakid species from Antarctic waters recorded significantly high values of intraspecific genetic variability in *Contracaecum osculatum* sp. D and *C. osculatum* sp. E, with high infection rates observed in their paratenic (icefish) and definitive (the Weddell seal, *Leptonychotes weddellii*) hosts, surpassing those found in sibling species of the *C. osculatum* (s.l.) complex from the Arctic Seas (Mattiucci and Nascetti, 2008). In addition, a 3-decade temporal analysis found stable values in both infection levels and genetic variability in these Antarctic anisakids (Mattiucci *et al.*, 2015*b*, 2017*b*). This trend of stability can be attributed to an undisturbed Antarctic marine habitat, fostering stable trophic webs with abundant hosts maintaining high anisakid population sizes, with less probability of genetic erosion events in their gene pools (Mattiucci *et al.*, 2015*b*, 2017*b*; Palomba *et al.*, 2023*b*).

In contrast, possible occurrence of genetic erosion was reported in a Mediterranean population of *A. pegreffii* after a 20-year (1986–2004) study. There was a loss of rare alleles at various polymorphic loci (i.e. allozymes) (Mattiucci and Nascetti, 2008). This was attributed by the authors to a likely demographic decline of the parasite, possibly due to habitat disturbance and host species population disruption, consequently altering the trophic web required by the parasite to complete its life cycle. A similar genetic analysis was conducted in the Austral (New Zealand waters) populations of the same parasite species, showing instead a stable genetic polymorphism (Mattiucci and Nascetti, 2008). More recent investigations of the Mediterranean populations of *A. pegreffii*, based on a substantial number (*N* = 11) of highly polymorphic DNA microsatellite (SSRs) loci, originally developed for the *A. simplex* (s.l.) complex (Mattiucci *et al.*, 2019; Bello *et al.*, 2020), provided further support to the alarming decrease of genetic variability in this parasite. According to those SSR loci, preliminary estimates of the genetic variability at intraspecific level over a spatial scale level, in a large number of individuals (*N* ≈ 150), analysed for each Mediterranean and extra-Mediterranean sample of *A. pegreffii*, have indicated lower mean heterozygosity values (*He*) in the Mediterranean populations (on average, *He* ≈ 0.67), compared with the levels observed in parasite populations from an extra-Mediterranean area, such as New Zealand waters (on average, *He* ≈ 0.75). Similarly, intraspecific genetic diversity estimated in *A. pegreffii* at a polymorphic mitochondrial gene locus (i.e. mtDNA *cox2*) showed a much lower nucleotide diversity (*π*) and haplotype number (Nh) (*π* ≈ 0.0059; Nh = 52) in a population from the Adriatic Sea compared with that recorded in its extra-Mediterranean population from New Zealand (*π* ≈ 0.0102; Nh = 113) waters. These findings suggest that the Mediterranean populations of *A. pegreffii* are facing genetic erosion events, probably linked to an overall parasite demographic fluctuation. In contrast, a higher level of stability characterizes the trophic webs maintaining *A. pegreffii* life cycle in the Austral waters off New Zealand. This stability is likely maintained by a broader range and demography of suitable hosts in the Austral region, which in turn, permits the maintenance of higher *A. pegreffii* population size, as above mentioned, with lower probability of genetic drift phenomena in the population's gene pool. These observations once again suggest how vulnerable the Mediterranean ecosystem could be in the face of recent global changes and anthropogenic impacts, as suggested by the likelihood of genetic variability loss in parasite's gene pool.

The Mediterranean population of *A. physeteris* has also displayed a high level of genetic diversity estimated at mitochondrial gene level, with a Tyrrhenian sample showing a considerable number of haplotypes and nucleotide diversity values (Nh = 39; *π* ≈ 0.0086) (Cipriani *et al.*, 2022*b*). This finding seems to be positively correlated with the high parasitic load so far observed in the Mediterranean populations of physeteriids (Santoro *et al.*, 2018*a*; Mazzariol *et al.*, 2018). Despite the fact that comparative spatial and/or temporal genetic data are not available for this parasite, and that nuclear genes should be investigated, the genetic variability values and parasitic burden observed seem to indicate a stable trophic web which sustains the life cycle of *A. physeteris*.

Genetic studies of Mediterranean *S. sulcata* at 3 gene loci (i.e. ITS region of rDNA, mtDNA *cox1* and *cox2*) revealed an overall genetic similarity between parasite populations from Adriatic and Tyrrhenian waters, without geographical clustering of parasite from different basins (Marcer *et al.*, 2020). This could likely be the consequence of the long-span migrations recorded for the loggerhead sea turtles throughout the Mediterranean Sea (Marcer *et al.*, 2020). However, the existence of a certain number of haplotypes (Nh = 38) observed at the mtDNA *cox1* gene locus in the Mediterranean population of *S. sulcata* was estimated (our unpublished data). This value would be positively related to the parasite population size maintained by both juveniles and adult loggerheads. Although a spatial and temporal comparison of the genetic diversity of *S. sulcata* from extra-Mediterranean waters cannot currently be performed, these findings represent the base for future genetic comparisons. Such a comparison that may be feasible with the possible existence of a sibling species of *S. sulcata* from the Eastern China Sea off Japan, showing a high level of genetic differentiation from Mediterranean isolates, was recently suggested (Sata and Nakano, 2023). Unfortunately, only 2 sequences were deposited from GenBank from Eastern China Sea, making it impossible to infer genetic variability values. Further genetic studies, potentially adopting a multigenotyping approach, will unravel the population genetic structure and genetic diversity of *S. sulcata* (s.l.) worldwide, and even clarify the possible existence of sibling species within the taxon. This information, combined with the definitive host's phylogeographical data, will be useful to monitor the host migratory routes, foraging areas and nesting sites in Mediterranean waters.

A recent molecular epidemiological study from different geographical areas of the Mediterranean Sea and Atlantic waters revealed a substantial genetic variability for *G. adenoplusia*. Specimens of *G. adenoplusia* were obtained from the benthic shark (*E. spinax*) in the eastern and western Mediterranean, as well as the Atlantic Ocean (Isbert *et al.*, 2023). The study revealed a noteworthy intraspecific occurrence of numerous haplotypes (Nh = 17) with 18 polymorphic sites in the large subunit ribosomal DNA (28S rDNA) gene (Isbert *et al.*, 2023). Interestingly, in the Mediterranean, the parasite showed higher abundance in intermediate hosts sampled from the Balearic and Cyprus populations, also corresponding with the highest record of polymorphic sites and haplotypes (the latter from the Balearic Sea) (Isbert *et al.*, 2023). This observation supports the hypothesis that, even in the case of this heteroxenous cestode, genetic variability is positively correlated with the parasite population size at the local level. Indirectly, this could imply that in the Mediterranean Sea, the deep-sea food webs maintain a higher stability than pelagic realm. Concerning *Grillotia* spp., further genetic markers and additional sampling will be necessary to elucidate the genetic structure and intraspecific genetic variation of this taxa, to assess its host range and explore its utility as an indicator of deep-sea environment stability.

## TTHs as sentinels of sea temperature rise

The biogeographical range and infection dynamics of host–parasite associations involving the chosen TTHs and their hosts are not only determined by biotic factors, but also profoundly influenced by abiotic environmental parameters, such as sea water temperature and salinity. These are strongly impacted by climate changes. The Mediterranean Sea has shown a consistent increase of sea surface temperature (SST) from 1979 to 2003 (overall average increase of 1.4°C), particularly in the eastern part of the basin (MedECC, 2020). Indeed, it is defined as a ‘hot spot’ for climate change (Pastor *et al.*, 2018). Temperature increase and water-level rise can provoke changes in water circulation and variations in salinity, representing key environmental factors for the marine organisms. These changes present limiting factors for the egg hatching and free-living larval stages of parasites, but also influence the abundance of first intermediate hosts and the migratory routes for feeding and/or reproduction of both intermediate/paratenic and definitive hosts. For instance, the hatching of eggs and the free-living larval stage of each anisakid requires an optimal thermal range (Diez *et al.*, 2022; Gomes *et al.*, 2023). An increase in sea water temperature as recorded for the Mediterranean Sea (MedECC, 2020) could potentially impact their survival and lead to a shift in parasite distribution to areas with more favourable abiotic conditions. These phenomena could lead to a change in the fitness of certain parasite species in a determined marine area and alter its distribution in different intermediate and definitive hosts.

**Figure 4.**
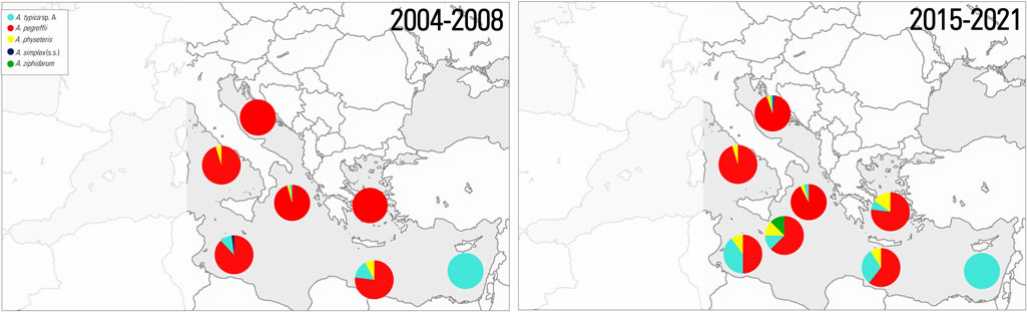
Relative proportions of *Anisakis* spp. from historical (2004–2008) and current (2015–2021) data observed in a fish species (i.e. European hake *Merluccius merluccius*) from different fishing grounds of the Mediterranean Sea (data from Mattiucci *et al.*, 2004, 2018*b*; Farjallah *et al.*, 2008; Mattiucci and Nascetti, 2008; Cipriani *et al.*, 2015 and unpublished data).

This is, for instance, the case in the parasite *Anisakis typica* (s.l.) whose distribution has been monitored over the past 2 decades in the Mediterranean Sea. Historically, this parasite showed a relative proportion of 100% in the European hake populations in the far eastern warmer Mediterranean Sea, i.e. around Cyprus waters and a relative proportion of around the 14 and 2.3%, in co-infection with other *Anisakis* spp. in hakes fished off Crete and from the Ionian Sea, respectively (Mattiucci *et al.*, 2004) (Fig. 4). In recent years, the occurrence of *A. typica* sp. A in this species from the eastern Mediterranean basin has increased, and it was also recorded at increasing proportions (around 25%) in Central Mediterranean (off the Malta coast) in co-infection with *A. pegreffii* and *A. physeteris* (Palomba and Mattiucci personal observation) (Fig. 4). In previous molecular epidemiological studies, this parasite was never reported from this basin (see Mattiucci *et al.*, 2018*a*, 2018*b*) (Fig. 4). Water temperatures in the Levantine, Aegean and northwest Ionian Seas have experienced the most significant SST increase in recent years (MedECC, 2020). In addition, warming was also recorded in central Mediterranean waters. This prompts speculation that the temperature increase could have contributed to the western-bound range shift of this parasite during recent years. Such temperature trends could increase egg hatching probability, thereby increasing the probability of transmission to the next suitable hosts. Further epidemiological surveys carried out in these fishing grounds may confirm this trend. Westward expansion of TTHs such as *A. typica* sp. A from the Levantine Seas to the southern Ionian and Central Mediterranean (Sicilian coasts) could serve as a further indicator of a warming Mediterranean Sea. The finding of *A. typica* sp. A in the eastern part of the Mediterranean Sea could be attributed to the so-called ‘Lessepsian migration’ of marine organisms, including parasites, from the Indian Ocean to the eastern waters of Mediterranean Sea through the Suez Canal. In a warmer Mediterranean basin, this parasite species could encounter environmental conditions resembling their current habitat, permitting to establish its life cycle.

Similarly, some recent findings indicate a potential northbound expansion of the geographical range of *A. pegreffii* into NE Atlantic waters. Previously limited to the northern part of the Atlantic Spanish coast, recent findings of its larval stages in the Atlantic cod, *Gadus morhua* and the Atlantic mackerel from the North Sea (Gay *et al.*, 2018; Levsen *et al.*, 2018*a*, 2018*b*) suggest a northward expansion. The lower limit of sea temperature tolerance of *A. pegreffii* along the Iberian Atlantic waters seems to be around 15°C (Diez *et al.*, 2022). Therefore, the ongoing rising sea temperature in NE Atlantic waters could permit the completion of *A. pegreffii* life cycle also in Northern regions. As a consequence, tracking the distribution of this parasite not only serves as an indicator of temperature shift in Atlantic waters but might also to predict the spread of this zoonotic parasite to other regions.

In the case of the loggerhead sea turtle, harbouring the anisakid *S. sulcata*, a westward movement of the host, likely due to SST increase, has been recently described (Mancino *et al.*, 2022). Thus, future data on the distribution and genetic analysis of *S. sulcata* can yield insights into turtle migration routes, foraging and nesting territories. Hence, monitoring the distribution and infection patterns of this chosen TTH would contribute to assessing the status of the loggerhead sea turtle and the trophic web within which *S. sulcata* completes its life cycle. Thus, this TTH could serve as a valuable indicator for monitoring this flagship host and the associated coastal and marine ecosystems, under climate change scenarios.

Temperature could also directly impact the eggs, coracidia and procercoids of trypanorhynch tapeworms (Sakanari and Moser, 1985). A notable example was reported by MacKenzie (1987). The author reported a dramatic decline in cestode populations, i.e. *Grillotia smarisgora* (Wagener, 1854) and *Lacistorhynchus tenuis* (Van Beneden, 1858) Pintner, 1913 in 2 fish hosts (Atlantic mackerel and the Atlantic herring *Clupea harengus*) in European waters over 8 years. The exact reasons behind these transmission failures remain puzzling; however, they are likely linked to shifts in abiotic environmental factors that could have affected the demography of the first intermediate host of the parasites. Intriguingly, the decline in cestode prevalence coincided with the conclusion of a North Atlantic hydrographic phenomenon known as the ‘1970s salinity anomaly’ (MacKenzie, 1987). Unfortunately, the Mediterranean Sea region lacks analogous reported instances in cestodes due to the scarcity of historical data for these parasites. Nonetheless, in recent years, a high rate of infection with trypanorhynch larvae such as species of genera *Grillotia*, *Molicola* and *Gymnorhynchus* in their intermediate hosts – not previously recorded – has been observed (e.g. Santoro *et al.*, 2018*b*, 2021*a*, 2022*b*; Palomba *et al.*, 2021*b*) (Fig. 3) (Supplementary Table 2). It could be speculated that the changes observed over the last years in Mediterranean waters (increase in SST, salinity, switching currents) could have altered the conditions for the survival and spread of these parasites. These changes could have triggered egg hatching, increased the abundance of the first intermediate hosts or potentially influenced the migration routes, food availability and reproductive cycles of their definitive elasmobranch hosts. Thus, changes in the abiotic conditions of Mediterranean Sea may have facilitated life cycle completion in these cestodes.

Furthermore, shifts in the geographic distribution of definitive and intermediate hosts of certain helminth parasites, driven by global climate changes, could create new parapatric and sympatric areas between isolated but genetically related parasite species. This could lead to a potential increase in hybridization events between closely related species. An example of this phenomenon is observed in the sympatric area of the Alboran Sea, where the 2 sibling species, i.e. *A. simplex* (s.s.) and *A. pegreffii*, coexist (see Mattiucci *et al.*, 2018*b*). Preliminary results obtained by a multinuclear genes approach (Mattiucci *et al.*, 2016, 2019; Palomba *et al.*, 2020 and unpublished data) have shown a differential distribution of hybrid genotype categories at the respective temperature tolerance limits of the 2 parental taxa within their overlapping geographic areas, such as the Alboran Sea waters and the Iberian Atlantic coast (Mattiucci *et al.*, 2022 and unpublished data). Hypotheses about the possible enhanced fitness of hybrid genotype categories with respect to the parental taxa remain speculative but cannot be ruled out. Thus, monitoring the distribution of *A. pegreffii* and *A. simplex* (s.s.) and their hybrid genotype categories in these sympatric areas will constitute valuable predictive metrics for tracking climate change in these peculiar oceanographic basins.

## TTHs as sentinels of environmental pollution

The Mediterranean marine environment receives a plethora of contaminants from both anthropogenic activities at sea and coastal areas affected by inland agricultural, industrial and urban sources. These pollutants can undergo biomagnification within marine food webs, leading to increased concentrations at higher trophic levels, depending on the feeding habits of predators (Sures and Nachev, 2022; Sures *et al.*, 2023).

One interesting aspect of the interaction between some TTHs endoparasites and pollutants is the positive effect related to the presence of certain endoparasites, which seem to be able to mitigate some pollutant stressors for their host species. Indeed, the capacity of cestodes – and a lesser extent of nematodes – to accumulate pollutants in their tissues is well known, at concentrations higher than those found in the host's tissues (Sures and Nachev, 2022). This supports the evidence that infected hosts may experience less severe effects of pollutants compared to non-infected hosts (Sures and Nachev, 2022).

In this context, trypanorhynch cestodes, i.e. *C. gracilis* merocercoid larvae, infecting the flesh of Mediterranean fish from Egypt, were analysed for heavy metals (Cu, Pb and Cd) using atomic absorption spectrophotometry. The results indicated significantly higher concentrations of heavy metals in the parasite tissues compared with the muscles of its fish host. The study concluded that *C. gracilis* larvae could be used as bio-indicator of heavy metal pollution, adding further evidence to the usefulness of parasites in monitoring environmental impacts (Mahmoud *et al.*, 2015).

A positive correlation between high abundance levels of parasites and microplastics has been recorded in European anchovies and the European pilchard from western Mediterranean waters (Pennino *et al.*, 2020). The authors noted that the Gulf of Lions, which showed the highest frequency of ingested microplastics, also had a higher infection rate with *Anisakis* in sardine and anchovies. These findings could be linked to the fact that both *Anisakis* spp. and microplastic are bioaccumulated in the fish host during the fish life history, through predation and feeding. However, a specific synergic effect between parasites infection and microplastic was not described.

Concentrations of mercury and polychlorinated biphenyls were also measured in the European hake infected with *Anisakis* larvae (Mille *et al.*, 2020). The authors found a positive correlation between the parasitic burden with *Anisakis* spp. and the methylmercury contamination in hake. This suggests that the presence of methylmercury can weaken the host's immune system enough to affect parasite infection intensity (Mille *et al.*, 2020). Indeed, it has been supposed that pollutants such as heavy metals and PCBs can influence host–parasite interactions (Marcogliese and Pietrock, 2011; Sures and Nachev, 2022). This, in turn, could exacerbate the pathological effect provoked by the parasite species on the host, whose immune response capacity may be compromised due to a particular pollutant, perhaps with a concomitant decline in its local population. Generally speaking, TTHs do not seem to have a pathogenic impact on their definitive hosts, likely due to the result of long co-evolutionary host–parasite adaptations. However, there are instances where TTHs have been linked to pathological conditions. For example, gastric lesions caused by adult *A. simplex* (s.l.) complex specimens have been reported in stranded delphinids from eastern Mediterranean waters (Hrabar *et al.*, 2017). However, the possibility of a synergistic pathological effect due to the presence of environmental pollutants and these endoparasites cannot be ruled out. Further investigation including experimental infection studies, using also additional innovative methodologies, is needed.

## Conclusions and future directions

This comprehensive review represents an update on the biodiversity and ecology of chosen TTHs occurring in the Mediterranean Sea, as well as the first attempt to use them to detect the consequences of anthropogenic-driven impacts on marine food webs. The findings so far acquired on the distribution and parasitic burden of some TTHs underscore that marine ecosystem stability, with stable food webs and abundant host populations, is associated with large parasitic populations. Consequently, the overall abundance of parasites and the genetic variability of specialized multi-host parasites, such as some TTHs here considered, can reflect the state of trophic webs in the Mediterranean marine ecosystem.

However, in some cases, we have detected that the population size of some TTHs (for instance, *A. pegreffii*, *H. corrugatum* and *H. incurvum*) is lower with respect to other extra-Mediterranean populations. These findings suggest that, in comparison with other oceanographic areas, the trophic webs involving hosts through which those parasites complete their life cycles in the Mediterranean are somehow altered. Probably, the Mediterranean Sea, being an enclosed and vulnerable ecosystem, is rapidly responding to ongoing global environmental changes, and is thus less resilient to these stressors. However, paradoxically,a high parasite burden suggests a well-preserved and functioning ecosystem.

The findings also suggest that the metrics here proposed, i.e. estimates of genetic variability and infection rates of parasites, could serve as indicators for monitoring the status of diverse marine trophic webs and, more broadly, the ‘health state’ of the Mediterranean Sea, over both temporal and spatial scales. In the light of current global anthropogenic changes, it will be advisable to study their effects also on the alteration of intraspecific genetic diversity, since this represents the basis for any evolutionary change, and it is a fundamental level of biodiversity. Despite the increasing number of studies examining the effects of global changes on the biodiversity of parasite species (Fiorenza *et al.*, 2019), very few studies focus on genetic diversity and are eventually related to the terrestrial ecosystem relevance (Gagne *et al.*, 2020; Gupta *et al.*, 2020). Future studies that will incorporate the intraspecific genetic variability of TTHs into investigations related to anthropogenic stressors will offer novel integrated avenues. These studies include the possibility to analyse population genetic structure based on genome-wide diversity, such as by dd-RADseq analysis, and genotyping individuals at climate-adaptive loci from different geographical populations. These investigations will also be possible with increased availability of genomic and transcriptomic resources for chosen TTHs (e.g. Cavallero *et al.*, 2018; Palomba *et al.*, 2022, 2023*b*). These studies will enable evaluation of the evolutionary potential of parasite species, populations and genotypes also in the context of global change scenarios.

Recent insights emphasize that parasites with multiple obligate hosts could face a higher risk of extinction in a given geographical area due to potential losses or decreases in suitable hosts, leading to bottleneck events in their life cycle (Carlson *et al.*, 2020; Sures *et al.*, 2023). This situation has been observed among the TTHs in the Mediterranean Sea. Stranding events of cetaceans, sea turtles and sharks in the Mediterranean Sea can occur for several reasons, including natural causes such as illness, environmental factors, as well as anthropogenic causes including bycatch, vessel strikes, plastic ingestion or acoustic trauma. In this context, the General Fisheries Commission for the Mediterranean (GFCM) has adopted several recommendations aimed at preserving these species. For instance, GFCM/42/2018/2 and GFCM/44/2021/16 propose mitigation measures for the conservation of teleostean and elasmobranch species in the Mediterranean. These recommendations support data collection and monitoring of these vulnerable ‘flagship species’. Consequently, obtaining insights not only into threatened ‘flagship’ host species, but also on the endoparasites they harbour becomes crucial for predicting variations in distribution, population sizes, migration patterns and health status of ‘flagship’ host species in response to environmental changes, such as rising sea temperatures and pollutants. The joint effect of contaminant biomagnification and parasites in definitive host species of the chosen TTHs from the Mediterranean Sea remains poorly studied. Future transcriptomic investigations, focusing on stressor-exposed host conditions, potentially in comparison with co-infecting TTH parasites, may elucidate the pathogenic pathways modulated during parasite and pollutant exposures.

Finally, considering the observed trends of increasing SSTs in the Mediterranean, there is evidence that global warming is also impacting the distribution of some parasite species. The anisakids *A. typica* (s.l.) and *S. sulcata*, well adapted to warmer water temperatures of the eastern Mediterranean basin, are both showing a westward expansion, achieving higher parasitic loads in definitive and intermediate hosts in the warming waters of central Mediterranean Sea. Therefore, these TTHs might serve as ‘sentinels’ for tracking climate changes. Overall, it appears fundamental to apply an integrative approach in future studies to gain a comprehensive understanding of stress effects on TTHs to monitoring changes in the dynamics of host–parasite associations of the marine Mediterranean ecosystem.

## Supporting information

Palomba et al. supplementary material Palomba et al. supplementary material

## Data Availability

Data availability is not applicable to this review article.

## Supplementary material

The supplementary material for this article can be found at https://doi.org/10.1017/S0031182023000951
