# Supplementary material for: An update and ecological perspective on certain sentinel helminth endoparasites within the Mediterranean Sea
Source: Parasitology. 2023 Oct 13;150(12):1139–57. doi: 10.1017/S0031182023000951 (PMC10941224; doi:10.1017/S0031182023000951)
Supplement: Palomba et al. supplementary material [file S0031182023000951sup001.doc]

| **Suppl. Table 1.** Trophic web transmitted helminth nematodes of mid-upper organisms in the Mediterranean Sea. Locality is reported according to the FAO major fishing areas (37) of the Mediterranean Sea. | | | | |
| --- | --- | --- | --- | --- |
| **Taxa** | **Locality** | **Intermediate/paratenic host** | **Definitive host** | **References** |
| **Rhabditida** |  |  |  |  |
| **Anisakidae** |  |  |  |  |
| *Anisakis pegreffii▲* | 1.1, 1.2, 1.3, 2.1, 2.2, 3.1, 3.2 | Trichiuridae, Zeidae, Scombridae, Carangidae, Gadidae, Mullidae, Xiphiidae, Merlucciidae, Engraulidae, Myctophidae, Sphyraenidae, Sparidae, Moronidae, Muraenidae, Phycidae, Trachinidae, Scyliorhinidae, Scorpaenidae, Ommastrephidae | *Grampus griseus, Stenella coeruleoalba, Tursiops truncatus, Kogia sima*, Physeter macrocephalus*, Ziphius cavirostris* | see Mattiucci *et al.,* 2018b and references therein; Cammilleri *et al.,* 2020; Pekmezci, 2021; Roca-Gerones *et al.,* 2021; Caballero-Huertas *et al.,* 2022; Cipriani *et al.,* 2022b; Santoro *et al.,* 2022c; Aydin and Pekmezci, 2023 |
| *A. typica▲* | 3.2, 2.1., 2.2 | Merlucciidae, Scombridae, Gadidae, Scorpaenidae, Phycidae, Trichiuridae | *Stenella coeruleoalba* | see Mattiucci *et al.,* 2018 and references therein; Mostafa *et al.,* 2020; Pekmezci and Onuk, 2020; Aydin and Pekmezci 2023 |
| *A. ziphidarum* | 1.1, 1.3, 2.2, 3.1 | Merlucciidae, Myctophidae, Scorpaenidae | *Ziphius cavirostris* | see Mattiucci *et al.,* 2018b and references therein; Cipriani *et al.,* 2022b |
| *A. simplex* (s.s.) | 1.1, 2.2, 2.1, 1.3 | Carangidae, Clupeidae, Engraulidae, Gadidae, Molidae, Merlucciidae, Scombridae | *Tursiops truncatus, Stenella coeruleoalba* | see Mattiucci *et al.,* 2018b and references therein; Cammilleri *et al.,* 2020; Mostafa *et al.,* 2020; Santoro *et al.,* 2020b; Meghanawy *et al.,* 2021 |
| *A. physeteris▲* | 1.1, 1.3, 2.1, 2.2, 3.1, 3.2 | Histioteuthidae, Ommastrephidae, Carangidae, Congridae, Lampridae, Trichiuridae, Etmopteridae, Gadidae, Merlucciidae, Myctophidae, Scombridae, Phycidae, Phosichthyidae, Scyliorhinidae, Xiphiidae | *Kogia sima, Physeter macrocephalus,* *Ziphius cavirostris* | see Mattiucci *et al.,* 2018b and references therein; Roca-Geronès *et al.,* 2020; Palomba *et al.,* 2021a; Cipriani *et al.,* 2022b; Santoro *et al.,* 2022c |
| *Sulcascaris sulcata▲* | 1.3, 2.1, 3.2 | *Pecten jacobaeus, Argopecten opercularis, Mytilus galloprovincialis, Bolinus brandaris* | *Caretta caretta* | Sey *et al.,* 1977; Manfredi *et al.,* 1998; Santoro *et al.,* 2010, 2019, 2020a; Gračan *et al.,* 2012; Marangi *et al.,* 2020; Gentile *et al.,* 2021; Marcer *et al.,* 2020; Pretto *et al.,* 2020 |
| **Raphidascarididae** |  |  |  |  |
| *Hysterothylacium aduncum* | 1.1, 1.3, 2.1, 2.2, 3.2, | *Sardina pilchardus, Sparus aurata, Diplodus vulgaris, Solea solea, Phycis phycis, P. blennoides, Rhinobatos rhinobatos, Trachurus trachurus, T. mediterraneus, Merluccius merluccius, Pagellus erythrinus, Sprattus sprattus, Engraulis encrasicolus, Micromesistius poutassou, Xiphias gladius, Alosa fallax, Arnoglossus laterna, Lophius piscatorius, Belone belone, Dicentrarchus labrax, Atherina boyeri, Arnoglossus rueppelli, Spicara smaris, Trisopterus minutus, Serranus cabrilla, S. hepatus, Boops boops, Aspitrigla obscura, Eutrigla gurnardus, Trigloporus lastoviza, Trigla lucerna* |  | Farjallah *et al.,* 2006; Genc *et al.,* 2005; Rello *et al.,* 2009; Kalay *et al.,* 2009; Amor *et al.,* 2011; Keskin *et al.,* 2015; Roca-Geronès *et al.,* 2020 |
| *Hysterothylacium corrugatum▲* | 1.3 |  | *Xiphias gladius* | see Mattiucci *et al.,* 2014 and references therein |
| *Hysterothylacium incurvum▲* | 1.3 |  | *Xiphias gladius* | see Mattiucci *et al.,* 2014 and references therein |
| *Hysterothylacium petteri* | 1.3 |  | *Xiphias gladius* | see Mattiucci *et al.,* 2014 and references therein |
|  |  |  |  |  |
| **Physalopteridae** |  |  |  |  |
| *Proleptus obtusus* | 2.2 |  | *Scyliorhinus canicula* | Gangemi *et al.,* 2019 |
|  |  |  |  |  |
| **Tetrameridae** |  |  |  |  |
| *Crassicauda grampicola* | 1.1 |  | *Grampus griseus* | Cuvertoret-Sanz *et al.,* 2020 |
|  |  |  |  |  |
| **Pseudaliidae** |  |  |  |  |
| *Halocercus delphini* | 1.1 |  | *Stenella coeruleoalba, Delphinus delphis* | Pool *et al.,* 2020 |
|  |  |  |  |  |

*immature parasite;*▲*species chosen as ecological indicators
